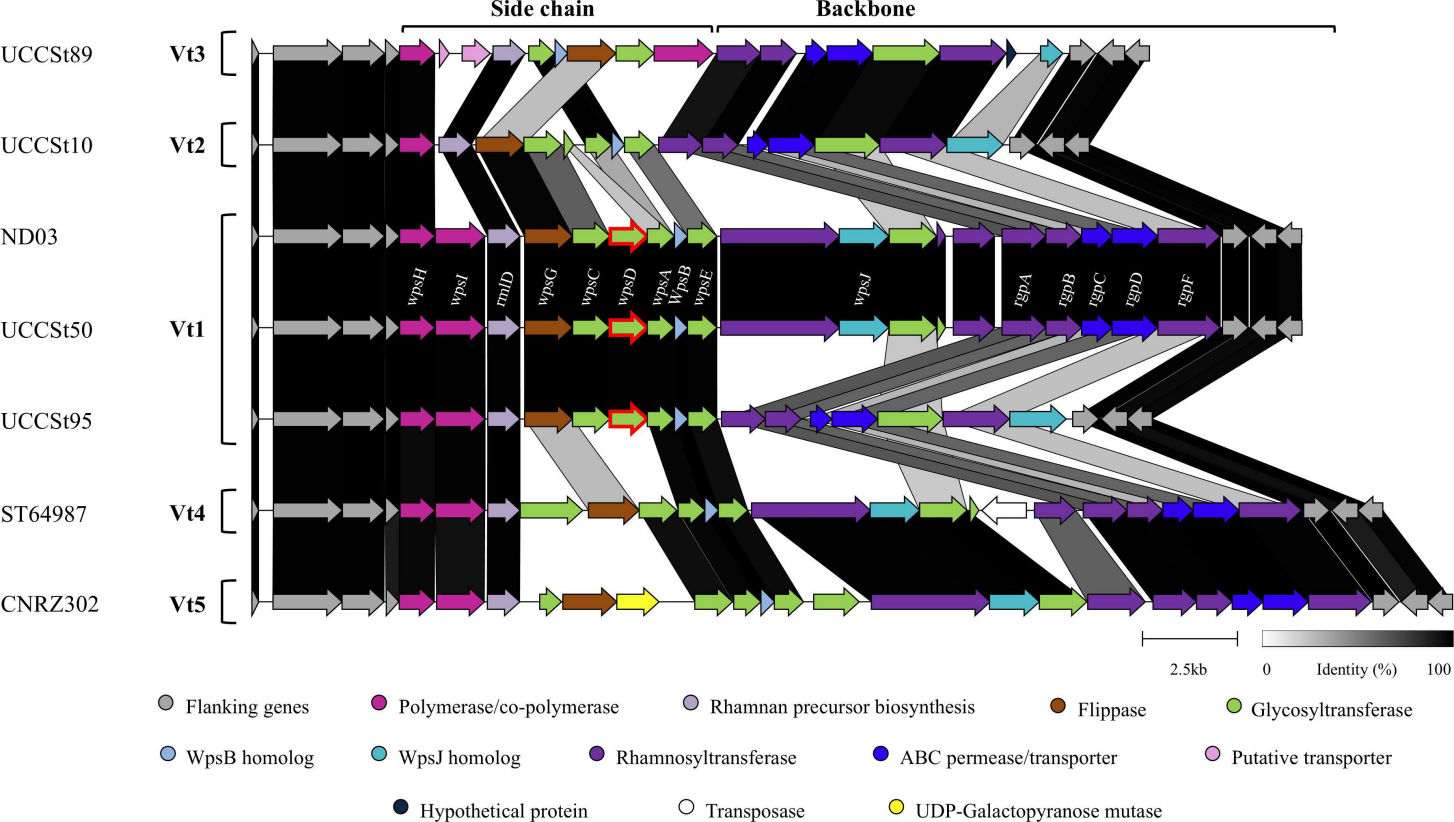# Articles of Significant Interest in This Issue

**DOI:** 10.1128/aem.02025-25

**Published:** 2025-10-22

## A SPOTLIGHT ON AEM’S PLANETARY MICROBIOLOGY SPECIAL COLLECTION

Guest editor Betül Kaçar (e00241-25) guides us through key article contributions in AEM’s Planetary Microbiology collection, highlighting the transformative microbial innovations and singularities making Earth what is today. (*Photo credit: ESO/Babak A. Tafreshi, Wikimedia Commons CC-BY 4.0*.)


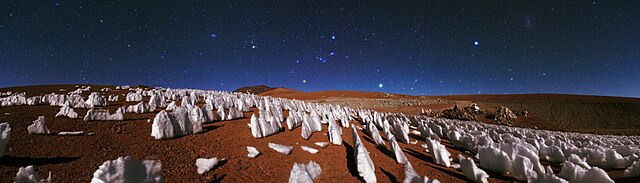


## SPOOKY VIROMES DURING CADAVER DECOMPOSITION 

Yu et al. (e01453-25) used machine learning to identify stage-specific viral succession patterns during the decomposition of buried animal remains. By regulating bacterial lysis, the viruses control postmortem microbial succession and serve as forensic biomarkers. 


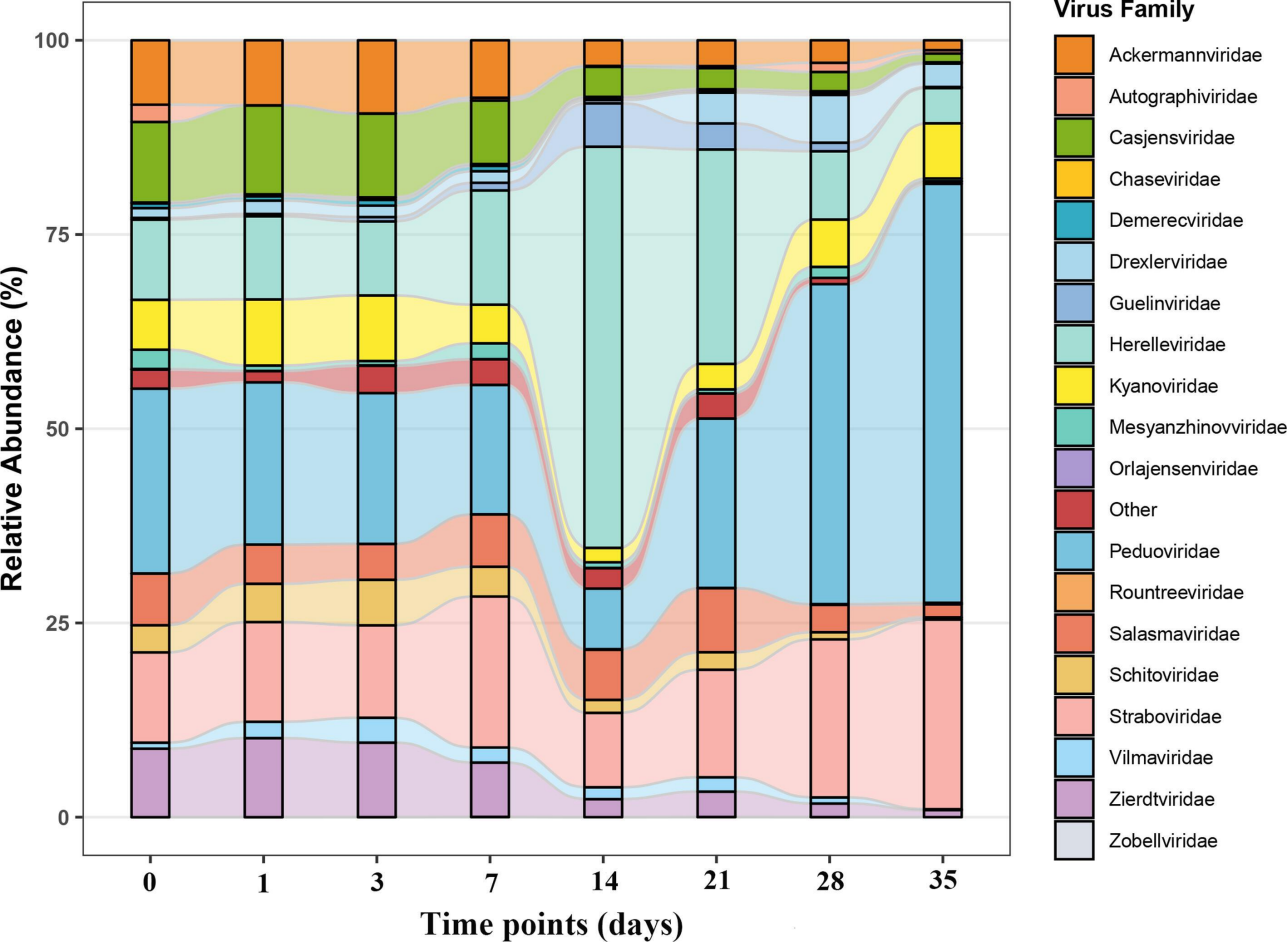


## BACTERIOPHAGE ENDOLYSINS IN THE FIGHT AGAINST ACNE 

Lee et al. (e01168-25) demonstrate the antibacterial activity of a peptide generated from the endolysin of anti-*Cutibacterium acnes* phages. The selectivity of the treatment against *C. acnes* underscores its potential to advance anti-acne therapeutics and other cosmetic applications.


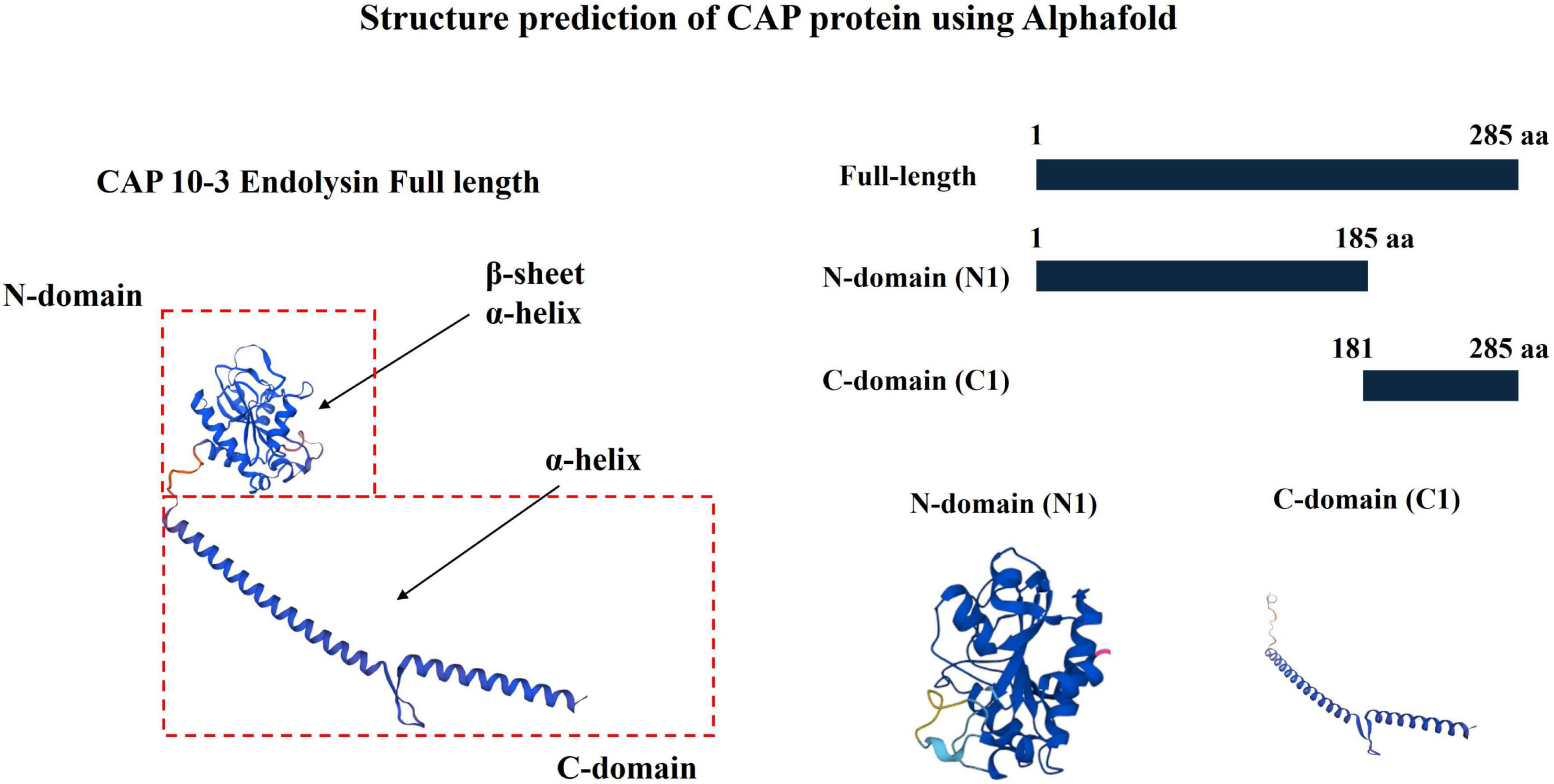


## ACIDOPHILES, SULFATE MINERALS, AND MARS

Gypsum and other sulfate deposits commonly found on Mars formed under acidic conditions early in the planet’s history. In this latest addition to AEM’s Planetary Microbiology collection, Havlena et al. (e01397-25) show that similar conditions in sulfuric caves support the activities of extremophilic microorganisms – driving the precipitation of the sulfate minerals from hydrogen sulfide gas.


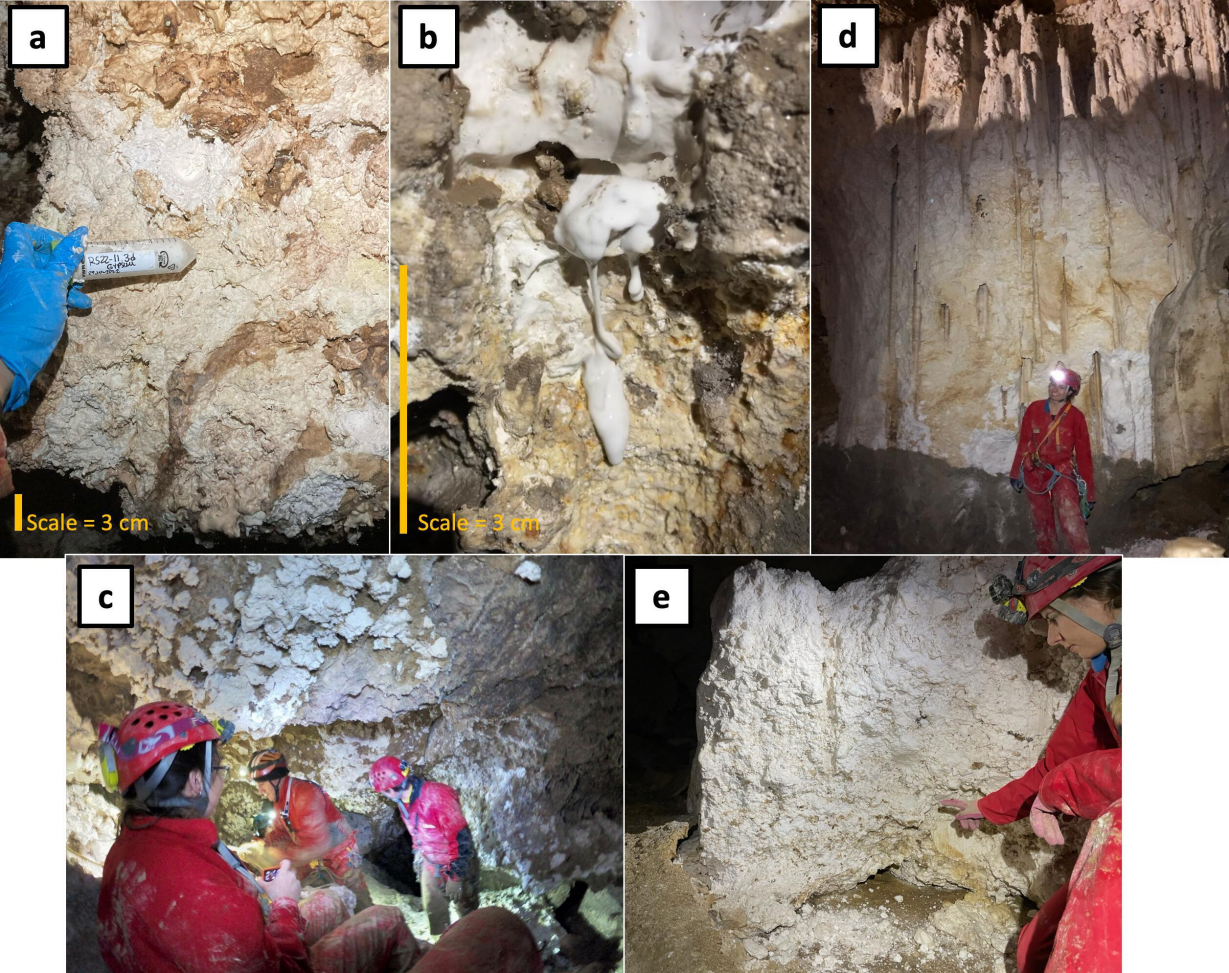


## BIOREMEDIATION TO THE RESCUE FOR MEGADIVERSE COUNTRIES 

Over 70% of the planet’s terrestrial biodiversity comes from megadiverse countries, many of which face widespread hydrocarbon and wastewater pollution. Morales-Mancera et al. (e01442-25) review sustainable, nature-based solutions to restore these environments and preserve their invaluable biodiversity. 


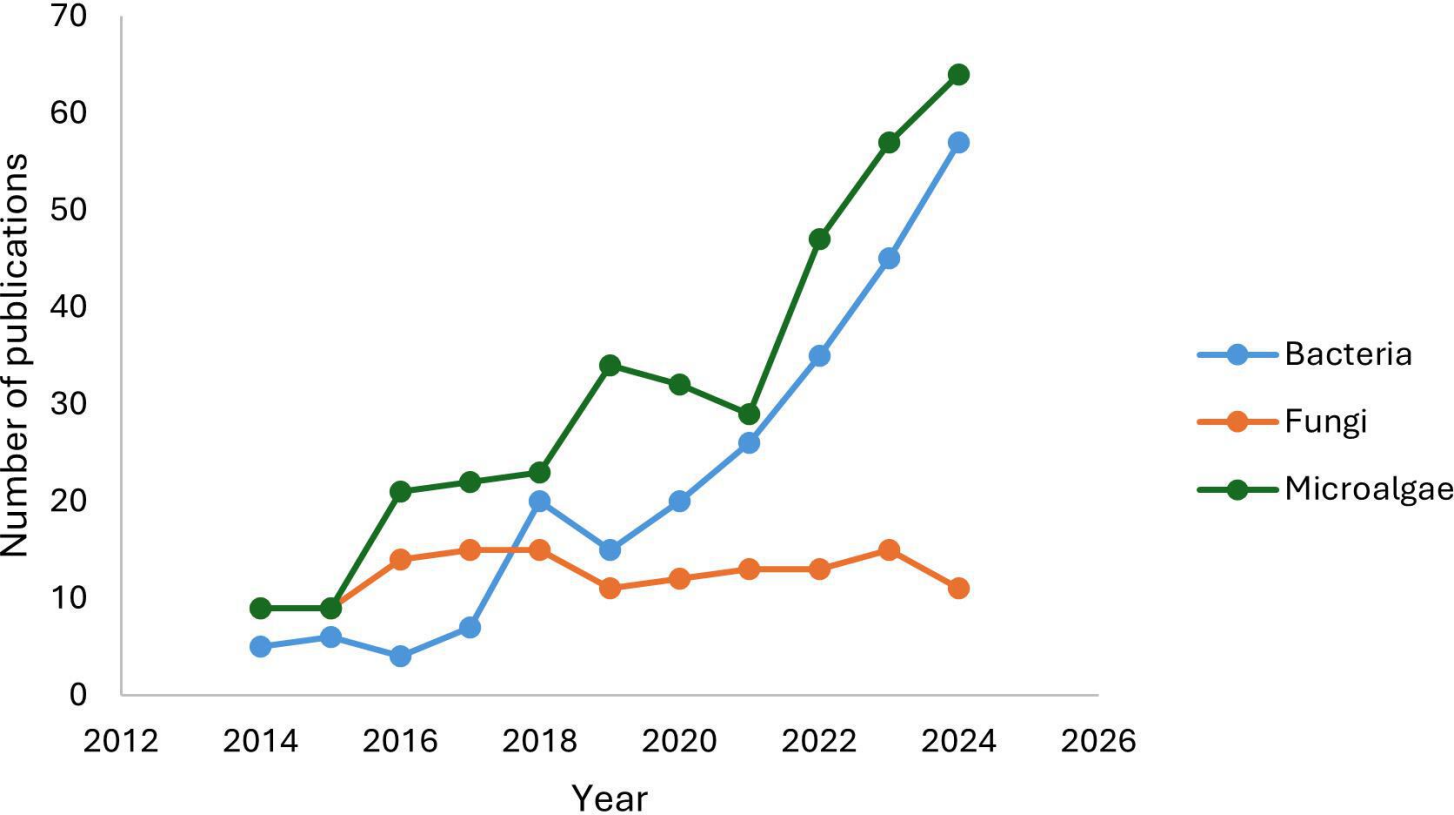


## OF CHEESE, YOGURT, AND PHAGES 

*Streptococcus thermophilus* is extensively used as a lactic bacterial starter culture in dairy fermentations threatened by bacteriophage infections. Kampff et al. (e01238-25) identified a surface receptor critical for phage-host interactions that can be targeted to develop more robust starter cultures.